# A qualitative study of the dynamics of access to remote antenatal care through the lens of candidacy

**DOI:** 10.1177/13558196231165361

**Published:** 2023-04-21

**Authors:** Lisa Hinton, Karolina Kuberska, Francesca Dakin, Nicola Boydell, Graham Martin, Tim Draycott, Cathy Winter, Richard J McManus, Lucy Chappell, Sanhita Chakrabarti, Elizabeth Howland, Janet Willars, Mary Dixon-Woods

**Affiliations:** 1 THIS Institute (The Healthcare Improvement Studies Institute), 2152 University of Cambridge, UK; 2 Usher Institute, The University of Edinburgh, UK; 3 4845 Royal College of Obstetricians and Gynaecologists, UK; 4 413474 PROMPT Maternity Foundation, Southmead Hospital, UK; 5 Nuffield Department of Primary Care Health Sciences, 6396 University of Oxford, UK; 6 Women and Children’s Health, 4616 King’s College London, St Thomas’ Hospital, UK; 7 Bedfordshire Clinical Commissioning Group, UK; 8 University Hospitals Birmingham, UK; 9 Department of Health Sciences, 4488 University of Leicester, UK

**Keywords:** Antenatal, remote care, candidacy

## Abstract

**Objective:**

We aimed to explore the experiences and perspectives of pregnant women, antenatal healthcare professionals, and system leaders to understand the impact of the implementation of remote provision of antenatal care during the COVID-19 pandemic and beyond.

**Methods:**

We conducted a qualitative study involving semi-structured interviews with 93 participants, including 45 individuals who had been pregnant during the study period, 34 health care professionals, and 14 managers and system-level stakeholders. Analysis was based on the constant comparative method and used the theoretical framework of candidacy.

**Results:**

We found that remote antenatal care had far-reaching effects on access when understood through the lens of candidacy. It altered women’s own identification of themselves and their babies as eligible for antenatal care. Navigating services became more challenging, often requiring considerable digital literacy and sociocultural capital. Services became less permeable, meaning that they were more difficult to use and demanding of the personal and social resources of users. Remote consultations were seen as more transactional in character and were limited by lack of face-to-face contact and safe spaces, making it more difficult for women to make their needs – both clinical and social – known, and for professionals to assess them. Operational and institutional challenges, including problems in sharing of antenatal records, were consequential. There were suggestions that a shift to remote provision of antenatal care might increase risks of inequities in access to care in relation to every feature of candidacy we characterised.

**Conclusion:**

It is important to recognise the implications for access to antenatal care of a shift to remote delivery. It is not a simple swap: it restructures many aspects of candidacy for care in ways that pose risks of amplifying existing intersectional inequalities that lead to poorer outcomes. Addressing these challenges through policy and practice action is needed to tackle these risks.

## Introduction

A striking and likely long-lasting impact of the COVID-19 pandemic has been increased use of remote forms of health care across many clinical areas. In England, the antenatal care pathway, which is designed to support women to achieve a successful and healthy pregnancy through a programme of regular monitoring and planned in-person visits, is an important example of this shift. In the English National Health Service (NHS), national recommendations^1^ and the exigencies of the pandemic resulted in large-scale conversion of many of the usual schedule of visits to remote contacts delivered by telephone or video from April 2020 onwards, with over 80% of antenatal appointments being conducted remotely from May to June 2020.^2^ As the pandemic continued through 2020–21, many aspects of antenatal care services that were formerly provided face-to-face remained remote in line with government policies, and some remote provision is likely to remain a long-term feature in the NHS.^3^

Studies examining remote care are often (rightly) concerned with outcomes.^4–7^ Questions of access have been more neglected yet they remain vitally important. In this study, we mobilise the construct of candidacy to explore remote antenatal care provision.^8^ Originally developed in the context of access to health care by vulnerable groups (Box 1), candidacy is a framework for understanding influences on access to health care. It recognises that access is jointly negotiated between the individual and the health service.^9^

Candidacy is characterised by seven features: identification of candidacy, navigation, permeability of services, appearances at health services, adjudications, offers and resistance, and operating conditions. These features can interact in multiple ways and are structured by socioeconomic, sociocultural, organisational, and institutional influences to determine access to care.

Only a small number of studies have used the candidacy construct to examine maternity care or women’s health^10,11^ and research on digital health and maternity care has remained very limited.^12^ Yet the distinctive features of antenatal care warrant specific attention, given its critical role in securing positive pregnancy outcomes, safeguarding women’s wellbeing and mitigating inequalities for under-served groups. Antenatal care is distinguished by the reality that most women and their babies remain well throughout pregnancy, but a range of high-risk concerns may emerge at any stage, and some may not be easily detected or assessed without specialist review by qualified professionals. The antenatal care pathway is designed to identify and respond to these risks through a highly structured system of routine monitoring and screening during this period, which also affords significant opportunity for unstructured interaction.^13,14^ But high quality antenatal care relies on many potentially precarious features of candidacy, including, for example, the ability of those who are pregnant to participate.^10^

**Box 1. Definition of candidacy**

‘[t]he ways in which people’s eligibility for medical attention and intervention is jointly negotiated between individuals and health services. … [Candidacy] is a dynamic and contingent process, constantly being defined and redefined through interactions between individuals and professionals, including how “cases” are constructed. Accomplishing access to healthcare requires considerable work on the part of users, and the amount, difficulty, and complexity of that work may operate as barriers to receipt of care. The social patterning of perceptions of health and health services, and a lack of alignment between the priorities and competencies of disadvantaged people and the organisation of health services, conspire to create vulnerabilities’.^8^

A further critical motivation for attention to antenatal care is the persistent and deeply troubling evidence of enduring inequities in maternal outcomes patterned by ethnicity and social disadvantage.^15^ In the United Kingdom, Black women (including those from African, Caribbean and other Black ethnic backgrounds), South Asian women, women of mixed ethnicity, and women with multiple disadvantages, such as a mental ill-health diagnosis or experience of domestic abuse, are overrepresented among the women who die during or up to one year after pregnancy.^16^ During COVID-19, the majority of women admitted to hospital were from Black or other visible minority ethnic groups and this trend continued to at least until April 2021.^17,^^18^

These challenges make urgent the need to understand the impact of the shift to remote provision on access to antenatal care. This study seeks to contribute to filling this gap through exploring the experiences and perspectives of pregnant women, antenatal health care professionals, and system leaders using the construct of candidacy.

## Methods

We conducted a qualitative study between September and December 2020 involving pregnant women, maternity staff, managers of maternity services and system-level stakeholders across the United Kingdom. Study participants were asked to discuss their experiences of receiving, providing, or organising antenatal care remotely during the COVID-19 pandemic.

### Sampling and recruitment

Recruitment strategies involved multiple routes, inviting participants via purposively selected NHS trusts, advertising through social media and professional and organisational networks, and working directly with community groups to maximise the diversity of the sample. NHS trusts were selected to ensure a range across geography, size of hospital trust and diversity of the population served. Social media was used to recruit women and staff. Routes included Twitter, professional and charity groups, and geographically targeted Facebook advertising. In the NHS trusts, posters publicised the study in maternity units. At a local level, community groups in two major cities supported recruitment of women from marginalised groups, including the Somali population and those experiencing poor mental health and isolation. Such groups could directly book interviews with willing women without the need for online access. Nationally, charities supporting women in pregnancy disseminated the study via their networks. These strategies ran throughout the study with additional approaches, such as working with health advocates in a diverse area of England, added in as recruitment progressed.

### Public and patient involvement

An expert collaborative group of 13 members was formed to provide advice and guidance on study design, data collection, and analysis. The group included ‘lay’ people who were (or had recently been) pregnant, health care professionals, system-level stakeholders, and representatives from charities. Collaborators advised on and helped with inclusive recruitment of participants. Interview topic guides were developed and piloted by the research team in discussion with the expert collaborative group.

### Data collection and analysis

All data were collected remotely due to the restrictions of the COVID-19 pandemic. Participants were able to access the participant information sheet online. In order to take part in an interview, they were asked to register on an online research platform (Thiscovery) to choose a mode of interview (telephone call or video call) and book a time slot. Health care staff, managers, and system-level stakeholders were also able to self-record answers to a set of questions asynchronously, in their own time without a researcher present. Participating pregnant women were offered a £25 shopping voucher. Consent was taken using an online form before the interview started. To mitigate the risks of digital exclusion, we offered the option of telephone-only participation for those who preferred this or did not have internet access. In these cases, consent was taken verbally by the researcher and entered into the online form. Data collection continued as analysis was progressed, with the sample size adapted to ensure diverse experiences were captured in line with the principle of information power.^19^

In-depth interviews were conducted by experienced qualitative researchers (LH, FD, KK, and JW) using semi-structured topic guides (see Online Supplement S1). The duration of the interviews ranged from 24 to 164 minutes. Interviews were audio-recorded for transcription and analysis. Transcripts were analysed by LH, FD, KK, and NB based on the constant comparative method, using initial open coding and the development of the coding framework, assisted by NVivo software.^20^ Researchers met regularly to discuss and amend the coding framework, which initially guided the data excerpt coding process, drawing on the constructs of candidacy to help structure the analysis.^8^

### Ethics approval

Ethics approval was granted by the NHS HRA West Midlands – Coventry and Warwickshire Research Ethics Committee on 22 July 2020 (20/WM/0204).

## Results

We interviewed 93 participants: 45 women who were pregnant or gave birth between March and September 2020 (indicated by the prefix ‘W’ in interview excerpts below), 34 health care professionals (prefix ‘H’), and 14 managers and system-level stakeholders, including representatives from national clinical bodies, pregnancy charities, and advocacy groups, six of whom were also practising health care professionals (prefix ‘M’) from across the UK. One interview involved two health care professionals together. Two further health care professionals who were interviewed had also received antenatal care during the study period and answered questions about both aspects of their experience.

The remote data collection method meant that we were unable to include participants without a reliable access to the internet or telephone, but we did achieve diversity of ethnicity in the sample of women interviewed; using Office for National Statistics classifications, 54% were White, 20% Black, 11% South Asian, 11% mixed ethnicity and 2% ‘other’, and 2% did not disclose their ethnicity.^21^ We did not collect data on ethnicity for other participants (Online Supplement S2). Table 1 provides details on women interviewed for this study.

**Table 1.** Characteristics of the service user subsample.

	Number of participants
First/subsequent pregnancy	
First time pregnant	21
Subsequent pregnancy	24
Total	45
Pregnancy outcome	
Live birth	14
Pregnancy loss	3
Pregnant at the time of the interview	28
Total	45
Mode of antenatal care	
Face-to-face and remote appointments	31
Mostly face-to-face appointments	10
Mostly remote appointments	4*
Total	45

Note. *Of these, three participants were between 19 and 21 weeks pregnant at the time of the interview, one participant was 9 weeks pregnant, and only had one appointment (booking).

Our analysis confirmed that the construct of candidacy offers a valuable way of understanding access to remote antenatal care, but we also found that it requires some adaptations to address both the specifics of the ‘two person’ nature of pregnancy and the distinctive character of the antenatal period. Six domains of the original candidacy framework were identified as particularly relevant: women’s identification of candidacy for themselves and their baby; navigation; permeability of services; appearing at services; adjudications; and operating conditions. A further domain in the original framework, namely, offers and resistance, was not identified as a particularly relevant feature for the remote antenatal care context.

### Women’s identification of candidacy for themselves and their baby

Antenatal care is characterised by a predefined schedule of visits once pregnancy is identified, but women are also expected to monitor any developments that might be indicative of complications, assess whether they are concerning, and activate help-seeking if needed. Complexity arises because, in antenatal care, candidacy assumes a ‘two person’ character, where individuals have to identify candidacy for both themselves and their baby, and this was not always straightforward.

I know people have to have responsibility for their own health and everything, but people don’t always, and it’s not just the mum is it, it’s the baby that’s involved as well? *(H21)*

Women’s accounts suggested that the shift to remote care impacted on the extent to which they saw themselves and their babies as eligible candidates for the attention of health services, both for routine appointments and for ad hoc issues that arose less predictably.

The feedback I received was that women were more likely not to engage when they had concerns, and therefore they [only] engaged when things got pretty bad. *(M12)*

Many women were, for example, apprehensive about being a nuisance or a burden on the NHS but were also worried about not knowing who to contact if they had concerns, such as reduced fetal movements. Sometimes they struggled to know whether and how to assert candidacy on behalf of themselves or their baby.

Because I always was wondering if I was missing something, should I have made an appointment, should I have done this, should I have done that? I wasn’t sure what the new schedule looked like, so I didn’t know whether I was on track, or I should have, you know, myself been proactive to put something in. *(W20)*

I don’t want to sound, like, paranoid just to call them and say, I’m really worried, I haven’t seen anyone because this is the recommendation, recommended, you know, plan from the NHS. And I’m not a professional so I thought this is probably adequate… I just tried to, like, keep calm. But it’s hard, you know, because you’re… Not wasting resource but just being… you know, you’re just being paranoid for nothing, don’t want to waste people’s time. *(W30)*

Many women expressed uncertainties in judging whether they could or should seek help, for example, in evaluating the significance of a missed clinical encounter, including a routine appointment, and whether they should insist on another one. Their confidence in assessing their eligibility for care was particularly influenced by previous experience of pregnancy and their expectations of and familiarity with antenatal services.

It’s been hard, but I don’t yet know what, you know… what I’m supposed to receive as it’s my first pregnancy, so I wasn’t really sure what to expect. *(W29)*

The midwife isn’t particularly chatty. I didn’t really want to, kind of, get into that stuff, or appear to be overly worried or, you know, so [laugh] paranoid about things. But, yeah, I did, sort of, start to wonder, like, you know, should I be buying my own blood pressure monitor, and using it, or do I not need to, or is there a certain point at which, you know, after twenty-something weeks I should be worried about my blood pressure, but not before that. So, yeah, all kinds of things like that which [laugh] if you start asking questions, I feel that you just come off as just being really panicky. *(W05)*

If women themselves did not identify their own candidacy (or their baby’s) for antenatal care, there were risks reported by stakeholders who were running support services that some, particularly those in more vulnerable groups, might not be picked up or referred.

But the women who would have self-referred anyway we saw, and we provided a service for them; but the women who would have been referred to us by health professionals, the health professionals hadn’t seen them to make those referrals anyway, so they just got completely lost in the system. And they’re the women that are turning up now really, really significantly poorly, somebody is actually picking them up now. And if they’d had an earlier intervention it wouldn’t have got this far. *(M02)*

### Navigation

Participants reported that the rapid reconfiguration of maternity services at the start of the pandemic had led to a proliferation of locally specific services and care pathways that added to the complexity of navigating the system. Information about the changes was dispersed across multiple channels, with no central or systematised approach to facilitate its use. Health care professionals described an increase in women not knowing whom to call; for example, a consultant midwife (with a management role) described their maternity assessment unit as ‘inundated with calls from women’ seeking information and guidance.

So what we found was that our maternity assessment unit was getting inundated with calls from women who didn’t know when they were going to be seen next, who was going to be leading on their care, how did they call their community midwife?” *(M14)*

Women often had to piece together information from multiple sources (e.g. direct contact with staff, calls or text messages to the community midwife, social media, health service websites, and formal guidelines), and some found that this added to their overall anxiety. Some women described making extensive use of digital resources to navigate the system. Those who were more technologically adept or who had more sociocultural capital, in terms of support, personal, or financial resources appeared to find it easier to access information or alternative forms of support, but even then, some struggled.

I tried to turn to my normal sources, either Reddit or Instagram, and depending where you are in the country it’s happening in a different way as well. So it feels like even if I’m looking for other sources, they don’t really clarify my situation about this particular hospital. *(W29)*

### Permeability of services

Some women and staff reported that remote appointments removed barriers to access and thus had potential to improve *permeability,* that is, the ease with which they could use services. For example, being able to save on transport costs and arranging childcare were identified by some as advantages of remote care.

Some mothers have said when you have multiple children and when they’ve been having to home school them and all those other things […] to be able to have a phone call or do those antenatal calls remotely actually has been easier for them. *(M01)*

However, permeability of services also deteriorated for some groups when antenatal care was provided remotely, including for those affected by material or social disadvantage. Analysis of staff and women’s reports identified risks of reduced permeability for those without access to the required technology to attend an online consultation, for example, as a result of digital poverty, limited technical literacy, or both. Staff raised concerns about women who did not own or have easy access to the devices needed to access video consulting platforms, lacked mobile data credit, or needed to pay for calls on a ‘pay-as-you-go’ basis.

There are many positives to it and it has certainly revolutionised a number of aspects of care… But I think we need to look at strategies for meeting the needs of our most vulnerable patients, which, inevitably means giving them smart phones, or the technology and the resources to be able to engage in this type of care, and to make sure that it’s not another change in practice that just meets the needs of people with privilege. *(H24)*

Using remote tools like phones or screens, which wasn’t easy actually because most of the mums that we work with experience poverty, and particularly re: digital poverty. So, they don’t necessarily have access to things like smartphones or tablets or computers. You know, they might not have the money required to get unlimited data. So, even doing like a video call would be probably out of the question for a lot of them. *(M11, support charity)*

Their husband might have a laptop or a smart phone but they don’t. *(M08)*

Less obviously, access to a private space at home where women felt able to speak freely was an important consideration for some. Maternity support workers assisting women from minority ethnic communities, including asylum seekers and refugees, highlighted the language and financial barriers faced by some women and the potential impacts of remote care on developing trust.

It has affected the trust, because when I used to carry out an antenatal group in the community, the women would attend there with an interpreter. They would become friends and they’d be able to gain our trust in order to tell us other issues they would be having. And that could be domestic violence, it could be other issues. It could be like they can’t afford items for the baby and things like that. And things like that can’t be done over the phone unless you’re together with that person. So, therefore, they were able to sort of build that relationship before and make sure it’s a safe place for them and it’s safe for them. *(H30 and H31; joint interview)*

### Appearing at services

Appearing at antenatal services involves women asserting a claim to candidacy for professional attention. We found that articulating such claims was complicated when appearances were remote. Both women and staff described the emotional and technical labour required to formulate, articulate, and assert their concerns via telephone or video calls that often felt rushed or tick-box-like. Participants found that some remote appointments felt hasty and lacking in opportunity for more informal, less protocol-driven interaction. This meant that there was little chance to discuss uncertainty, raise anxieties, or bring up issues that were not already on the clinician's agenda.

Women reported being apprehensive about staff not being able to see them in person and about not being able to accurately explain their pregnancy concerns to their health care professionals. Women also reported challenges in raising concerns about their mental health or asserting candidacy for emotional care, for example, in situations where difficult conversations about risks to their baby were required.

I suppose they were asking difficult questions about having an amniocentesis, and also they had to tell me over the phone that the risk to the baby is that the iron was depositing in my placenta and that was going to slow the growth. Those are two very different things but quite difficult things… […] But I suppose that’s the downside of the remote care. They’re both really big serious things to discuss with someone that you can’t see and that person has no idea how you cope with that conversation really because they can’t see you. *(W35)*

Appearing at remote services was further complicated by incomplete professional access to patient data systems. Maternity notes were regularly not joined up with the latest test results, and it was often the women, not the health professionals, who had the latest information in front of them, with no obvious way of sharing it with clinicians. Women therefore had to take administrative and technological responsibility for keeping track of their own notes and test results. They also were required to raise issues at remote consultations that would otherwise be raised by a health care professional with access to the notes or test results. The ability to meet these requirements is unlikely to be equally distributed.

They give you the folder at the beginning, and the midwife fills that in on paper, but then I think has an electronic record, which is… doesn’t like… might have more information in it. The scan reports were printed out and added to that, but then obviously there are things in my hospital record which are not in there. So, if you were to look at just the printed, you know… the thing that I have to take round with me, you definitely wouldn’t get a full picture of everything. […] But anyway, I keep a separate… […] a paper diary, where I just log all of my appointments, and test results that I’ve got access to and, so I can just keep track of everything in one place as well. *(W05)*

Where maternity notes were not easily accessible, women had to make more effort to assert their candidacy during interactions with maternity staff. Health professionals described the additional burden this placed on the remote consultation, and the shift of responsibility to women to share information in the notes they had at home and to flag any issues of concern.

Asking a woman to tell me what’s written in her notes and she doesn’t know where to find it. And it was difficult because not all the community midwives were recording those measurements into the patient record electronically, which meant it was difficult for hospital-based clinicians to see what was happening. *(H11)*

### Adjudications

Remote care impacted the way in which health care professionals were able to make judgements about the candidacy of a pregnant woman and her baby, with staff concerned that caring for women remotely hindered their assessment of their needs and wellbeing. Staff reported that they found remote identification of symptoms challenging, with visual signs like a rash or swollen ankles difficult to assess. While video calls might have helped to overcome these barriers, they were not universally available or reliable.

So it’s like I’ve got a funny rash or my skin’s gone a bit blotchy, you’re having to use words to describe it. So you have to say, well, you probably need to book in with the GP because somebody needs to see this, we can’t see over the phone. *(H20)*

They can be very vague, very non-specific and if they’re phoning up with a rash or… they’re trying to describe a vaginal discharge and you are trying to work out what they mean, it could just be an infection or is this normal, sometimes video calling would make it so much easier. You could just look and say, yeah, no, that’s normal, or, oh okay, yeah, I think we probably need to see you and take a swab. *(H28)*

Remote appointments were consistently described by both women and health care professionals as rushed and transactional, with telephone consultations being particularly limited by the loss of visual cues and clues. Staff described how the remote and impersonal nature of telephone and video appointments could undermine rapport, trust, and relationship building. Vital visual clues, including body language, were rendered invisible and could impede early identification and assessment of worrying symptoms or safeguarding issues.

You know, midwifery is a science, but it is also an art, and it relies on our being together and picking up on people’s communication skills, their… you know, their social situations, their body language, the relationships they have, you can’t pick that up on a video. *(H24)*

We had a couple of situations where women came on and we looked at them and thought, oh, my goodness, you’re really oedemic; we think you should come up, you know. And then they had pre-eclampsia or… so, I think that being able to visualise somebody, both from a relationship point of view, but also from a clinical point of view, just kind of reassures a little, compared to maybe a telephone call. So, you know, we are quite tight that if you can’t do video calls you do have to come in for face-to-face. *(H14)*

### Operating conditions and the local production of candidacy

Candidacy was seen, in its original formulation, to be influenced by local operating conditions that include the availability of resources and local pressures. Our analysis suggests that local information technology (IT) systems and technological capability influenced access to, and engagement with, antenatal care provided remotely. Health professionals described the negative impact of fragmented health records and IT systems on their care. They were frequently unable to access all the information they needed for a consultation or had to undertake ‘detective work’ across multiple systems. In some trusts, video consultation software did not interface with the maternity records. Staff frequently had to rely on women to provide the information from their maternity notes, with the adverse consequences for pregnant women appearing at services and health professionals feeling able to make satisfactory judgments about the candidacy of the pregnant woman and her baby, as noted above.

Additionally, remote appointments impeded the way relationships between women and health care professionals were established and sustained, which could further exacerbate the impact of the lack of continuity of care that some women found. Women were often left feeling an absence of personal connection without face-to-face interactions. The experience of remote services was better where there was continuity of care, but this was not universal. Many women never spoke with the same midwife twice. The absence of a relationship with a midwife sometimes meant women felt compelled to maintain a heightened level of vigilance to ensure they received necessary care.

So, the impact was the relationship really. Women have said to me a number of times, it’s really impacted their relationship and the ability for the midwives to pick up on issues that they would have potentially picked up on if they’d have met them at the beginning. So, I think it would, it’s really affected women’s relationships and their trust. *(H24)*

## Discussion

This large qualitative study suggests that careful attention is required to the implications for access of a shift to remote modalities for antenatal care. Using candidacy as a theoretical lens, we demonstrate that remote consultations are not a simple swap for face-to-face appointments. Instead, they impact antenatal care in complex and layered ways, mediated both by local technical and system capabilities and by the social and material resources available to those who are pregnant. Remote antenatal care can alter how women make judgments about their own care needs, complicating their ability to identify their own eligibility for health care or to make a claim for attention from the system. Remote forms of provision can change how easy it is to navigate care pathways and the permeability of services and can increase the effort and competencies required of women to ask questions, share information and flag up concerns. Remote consultations may also reshape the nature and quality of the relationships between maternity service users and staff, and impact on how clinicians evaluate and make judgements about care needs. Our study suggests that continuity of care, already problematic prepandemic, may be even more challenging to achieve remotely, despite its known benefits.^22,23^ Many of the challenges of candidacy in remote care are features of face-to-face care as well. But recognition of the particularities of what remote care does to candidacy for antenatal services enables fresh understanding of how its promises and risks might be better managed.

Our study showed that asserting and evaluating eligibility for professional attention was made more complex for pregnant women by the new tensions, frictions, and uncertainties introduced by remote care. Multiple factors may contribute to a woman identifying a concern about her own or her baby’s wellbeing, seeking and using antenatal services, and being able to receive the right care at the right time, but how remote care mediates these is not straightforward.

Remote care had important impacts on women’s ‘appearances’ at antenatal care and professionals’ ‘adjudications’ about the care needed. We found, as has been reported in other studies,^11^ that people may experience difficulties formulating and articulating information about their pregnancies and acting as an information source. This is especially problematic in situations where clinicians are having to rely more on service users when it comes to noticing and reporting symptoms, and where they do not always have ready access to complete records. These challenges may particularly affect nontransactional forms of care, including professionals’ ability to detect and assess new symptoms or to attend to social or domestic circumstances.

More generally, there was some evidence that candidacy for remote care may be patterned according to availability of social capital or material resources, raising concerns that those already disadvantaged could be further disadvantaged. Navigating and using antenatal care can be more convenient and efficient for some aspects of care and for some people when provided remotely, and for these groups it may enhance permeability. But it may also pose multiple additional barriers, and the effort required from women to engage with and use services can be significant. One challenge concerns the increased ‘responsibilisation’ implied by remote care models. Those less fluent in participatory approaches to health, less able to exercise responsibility and self-efficacy^24–27^ and lacking in socio-economic capital, including having a private space to take a call, a phone, and availability to be phoned, may be alienated or put at risk by the degree of responsibility they have to assume for their own antenatal health in remote appointments. Similarly, the use of digital resources described by some participants can help support understanding as to which symptoms require professional attention, but it requires ‘digital work’ that individuals are not equally able to undertake.^12^ In a context where women may not know what is expected of them in antenatal care and where socially disadvantaged women may lack knowledge or resources for digital technology, delays, and poor quality care may result.^28,29^

These are important concerns given the relationship between engagement with antenatal care and maternal and neonatal outcomes, making it crucial to pay attention to who is left out or left behind when care is provided remotely.^10^ Women with social risk factors, for example, those in poverty, victims or abuse or young mothers, are over 50% more likely to experience a stillbirth or neonatal death and carry a four-fold greater risk of maternal death.^10,16,30^ In the antenatal period, women are expected to take responsibility for their own health and for that of their baby, for example, by abstaining from risky substances such as alcohol and tobacco, and to identify any changes in their baby’s health, for example, reduced fetal movements.^31,32^ The dual nature of pregnancy, with its focus on the health of the woman and baby, and therefore two candidacies, and the continuous negotiations of care that are required throughout a woman’s maternity journey, render these considerations especially critical.^12,33^

### Strengths and limitations

This study involved a large number of participants including pregnant women, antenatal health care professionals, and system leaders for this type of research. The study was carried out during the autumn of 2020, before COVID vaccination was available, and during a time when remote antenatal care provision was at a very high level in the UK. While recognising that this context will have influenced the data, we have sought to direct our analysis towards enduring learning.

Bringing the lens of candidacy has allowed identification of overlap and interdependencies between the domains, and our analysis highlights the complex dynamics of introducing new care pathways and challenges an understanding of remote care as primarily beneficial. Conducting the study online allowed us to collect data during the restrictions imposed by social distancing requirements. Our inclusive recruitment strategies were successful in yielding an ethnically diverse sample (Online Supplement S2). We made efforts to ensure inclusive recruitment and offer alternatives to online participation, but were not able to directly include those without access to a telephone or those who had failed to get to antenatal care at all, although some of the concerns of such women were raised by specialist health care professionals and system leaders. Remote interviewing facilitated data collection during the pandemic, and the collection of a large, nationwide sample, but building rapport during a video or telephone interview was more difficult than it would have been face to face. This may have impacted the depth of the data collected.

### Implications for policy and practice

The 2022 Women’s Health Strategy for England gives welcome priority to tackling disparities in women’s health and barriers to access and experience of services, and sets out responsibilities for delivery.^34^ Other policies outline ambitions to exploit digital solutions and ‘lock in’ new approaches tried in the COVID-19 response.^35^ However, based on our findings, we argue that additional steps should be taken if these policies are to be successful. First, remote antenatal care services should be optimised for equality, inclusion and diversity and, critically, co-designed with maternity service users and representation from minoritised and marginalised groups to achieve this goal. Second, services should be designed in ways that are sensitive to digital exclusion and phone poverty. Third, policy and practice should consider whether the increased responsibilisation implied by remote antenatal care is suitable for all and ensure adequate alternative services are provided. Fourth, service development should be sensitive to the unintended consequences of remote care that may increase inequalities or clinical risks. Fifth, hybrid services might alleviate some of this risk and should therefore be considered as part of the solution, while ensuring that new models of care are evaluated. Sixth, women and their families should have access to information on the range of options for access and allow them to make an informed choice where possible; this requires systematic monitoring of how women are accessing information. Seventh, when in-person antenatal care is not universally available, service development should account for disadvantage in considering who should be prioritised for this form of care. Finally, there should be a reliable system for data collection relating to ethnicity and other consequential demographic characteristics, including regular analysis of their association with choices about service provision as well as outcomes, and use of these insights to inform ongoing improvement and redesign of services.

## Conclusions

Remote care might be here to stay and this has important implications for the dynamics of access. Our study suggests that remote care may restructure aspects of candidacy for antenatal care, with some of these posing risks of amplifying existing intersectional inequalities that lead to poorer outcomes. By exploring the impact of remote care pathways for both service users and professionals using the lens of candidacy, this study has identified a set of important challenges that will need addressing at policy and practice levels.

## Supplemental Material

Supplemental Material - A qualitative study of the dynamics of access to remote antenatal care through the lens of candidacy Click here for additional data file. Supplemental Material for A qualitative study of the dynamics of access to remote antenatal care through the lens of candidacy by Lisa Hinton, Karolina Kuberska, Francesca Dakin, Nicola Boydell, Graham Martin, Tim Draycott, Cathy Winter, Richard J McManus, Lucy Chappell, Sanhita Chakrabarti, Elizabeth Howland, Janet Willars, and Mary Dixon-Woods in Journal of Health Services Research & Policy
