# Supplementary material for: A qualitative study of the dynamics of access to remote antenatal care through the lens of candidacy
Source: J Health Serv Res Policy. 2023 Apr 21;28(4):222–32. doi: 10.1177/13558196231165361 (PMC10515462; doi:10.1177/13558196231165361)
Supplement: Supplemental Material - A qualitative study of the dynamics of access to remote antenatal care through the lens of candidacy [file sj-pdf-1-hsr-10.1177_13558196231165361.pdf]

Journal of Health Services Research & Policy

A qualitative study of the dynamics of access to remote antenatal care through the lens of candidacy

Hinton L, et al

Online supplement

S1. Topic guides

1 Women and their families

Background questions:

- How old are you?
- How many times have you been pregnant?
- What is the first part of your postcode?
- What ethnic group do you identify as?
- How did you hear about the study?

Current pregnancy status:

- Can you tell me a bit more about your current pregnancy?
- How far along are you **OR** when did you give birth?
- Have you been pregnant before?
- What was it like for you to receive antenatal care during the pandemic?

Antenatal experience:

- What antenatal care have you received so far? Which parts of your care were remote (i.e. provided by phone, email or video call) and what was face-to-face.
    - What was covered in these?
    - Did you feel anything was missing?
      (Chance to ask questions, feeling of being supported, opportunity to discuss worries, particular measurements, ability to physically show your care team something.)
- Has your partner been able to attend any/all of your appointments?
- How have physical checks been carried out?
- How many appointments have you had?
- Did you have any information about these changes ahead of time **AND** what information did you receive about how remote antenatal care was to be provided?
- How easy is it to get hold of your midwife or antenatal care provider remotely? Any problems encountered?
- Were you able to see/contact the same midwife in multiple appointments?
- If applicable: how does this experience compare to your previous pregnancies?
- Have you missed any appointments? Why?
  (Due to lack of transport, anxieties around being in public/ at a medical centre/ catching coronavirus?)
    - Were they remote or in-person?
- How easy is it to raise concerns remotely? What response have you had?
- What barriers have you come across?
- Can you tell me about your experience of attending antenatal classes?
    - Were they remote or in-person?

- Have you been able to hold a record of your antenatal care? What has that been like?
- Have you felt safe?
- How have remote consultations impacted on your relationship with your antenatal care professionals/providers?

Supplementing antenatal experience:

- Have you sought antenatal care and advice elsewhere?
  Searching online for information or reassurance, looking for external remote care, external in-person care (e.g. additional tests or ultrasound scans). To fill in gaps you've perceived in your antenatal care.
- Where have you sought support outside of NHS antenatal care?

What could change:

- What has worked well and what hasn't worked so well?
- What parts of your antenatal care do you think should be done in-person??
- What would improve your remote antenatal appointments?
- What would improve your in-person antenatal appointments?

What's important to you:

- Do you feel that current remote antenatal care addresses your needs?
- Have you felt any loneliness or isolation receiving remote care?
- Have you been taking any measurements, such as blood pressure, at home? If yes, please tell me about it. If no, would you feel comfortable to do that?
- How would you like information about remote antenatal care to be communicated to you?
  (E.g. leaflets, webpages, social media.)
- What would you include in remote antenatal care guidance for professionals?

Questions for birth partners/family members:

- What has your experience been? Have you been able to ask questions, raise concerns?
- Were you able to be involved in any antenatal classes?

Other issues:

- Imagine you're speaking with someone newly pregnant, receiving antenatal care at your hospital:
  - How would you describe your experience of antenatal care during the pandemic to someone who is newly pregnant?
  - What advice would you give them about receiving remote antenatal care?
- Is there anything else you wish to add about your remote and in-person antenatal care that we have not discussed?

2 Health care Professionals

2.1 Online live interview

(1) What is your job title?
(2) Please can you describe your current role and how it relates to antenatal care?
(3) Please describe how antenatal care has changed during the pandemic in your workplace?
(4) How was information about changes to antenatal care communicated to you? What information and communication would you have liked? Was there any training?
(5) Please describe how you have been providing antenatal care during the pandemic (in-person visits, telephone calls, video chats, email).
What was covered in these appointments?
(6) Please tell us about managing remote and in-person appointments, including scheduling and attendance. If there were cases of missed appointments, what was the reason given?
(7) Can you tell us about keeping paper or electronic records whilst delivering remote antenatal care?
(8) Have you had concerns about the safety or acceptability of remote antenatal consultations?
(9) How do you think the quality of antenatal care during the pandemic compares to that beforehand?
(10) What has worked well in remote antenatal care? Please give examples.
(11) What will you retain for future practice?
(12) What aspects of remote antenatal care don't work well or could be improved? Please give examples.
(13) How well do you feel that current remote antenatal care addresses the needs of the pregnant women you care for?
(14) Can you comment on how Black, Asian and Minority Ethnic and/or vulnerable women and families have been impacted?
(15) Are women able to disclose anything they need to during remote antenatal consultations?
(16) What are your thoughts about pregnant women doing some of their measurements at home as part of remote antenatal care?
(17) What would you include in guidance for healthcare professionals for remote antenatal care?
(18) Is there anything else you wish to add about remote antenatal care that we have not discussed?

2.2 On demand interview

(1) Please can you describe your current role and how it relates to antenatal care.
(2) Please describe how antenatal care has changed during the pandemic in your workplace?
(3) How was information about changes to antenatal care communicated to you? What information and communication would you have liked? Was there any training?
(4) Please describe how you have been providing antenatal care during the pandemic (in-person visits, telephone calls, video chats, email). What was covered in these appointments?

(5) Please tell us about managing remote and in-person appointments, including scheduling and attendance. If there were cases of missed appointments, what was the reason given?
(6) Can you tell us about keeping paper or electronic records whilst delivering remote antenatal care?
(7) Have you had concerns about the safety or acceptability of remote antenatal consultations?
(8) How do you think the quality of antenatal care during the pandemic compares to that beforehand?
(9) What has worked well in remote antenatal care? Please give examples.
(10) What will you retain for future practice?
(11) What aspects of remote antenatal care don't work well or could be improved? Please give examples.
(12) How well do you feel that current remote antenatal care addresses the needs of the pregnant women you care for?
(13) Can you comment on how Black, Asian and Minority Ethnic and/or vulnerable women and families have been impacted?
(14) Are women able to disclose anything they need to during remote antenatal consultations?
(15) What are your thoughts about pregnant women doing some of their measurements at home as part of remote antenatal care?
(16) What would you include in guidance for healthcare professionals for remote antenatal care?
(17) Is there anything else you wish to add about remote antenatal care that we have not discussed?

3 Managers and system-level stakeholders

3.1 Online live interviews

(1) What is your job title?
(2) Please can you describe your current role and how it relates to antenatal care?
(3) How has the provision of antenatal care in your service changed during the pandemic?
(4) What changes to the provision of antenatal care were decided by more senior colleagues, and how were these changes communicated to you?
(5) What changes to antenatal care were you involved in at a local level, and how did you communicate and implement these changes in your unit/trust?
(6) How (if at all) were service user groups such as MSLCs/MVP involved in decision making about these changes to antenatal care? Have you had any feedback from service users?
(7) Do you think any of these changes to remote antenatal care could have been improved?
(8) Did you create any local guidance? If so, please tell us about it.
(9) Have you noticed any impact from moving to remote antenatal care, and are these impacts being monitored?
(10) Do you have examples of what has worked well in remote antenatal care? What will you retain for future practice?
(11) What aspects of remote antenatal care don't work well or could be improved?
(12) How well do you feel that current remote antenatal care addresses the needs of pregnant women?
(13) Can you comment on how Black, Asian, or other Minority Ethnic groups and/or vulnerable women and families have been impacted?
(14) What are your thoughts about pregnant women doing some of their measurements at home as part of remote antenatal care?
(15) How well do you feel that current remote antenatal care addresses the needs of clinicians delivering care?
(16) What would you include in guidance for healthcare professionals for remote antenatal care?
(17) Is there anything else you wish to add about remote antenatal care that we have not discussed?

3.2 On demand interview

(1) Please can you describe your current role and how it relates to antenatal care?
(2) How has the provision of antenatal care in your service changed during the pandemic?
(3) What changes to the provision of antenatal care were decided by more senior colleagues, and how were these changes communicated to you?
(4) What changes to antenatal care were you involved in at a local level, and how did you communicate and implement these changes in your unit/trust?
(5) How (if at all) were service user groups such as MSLCs/MVP involved in decision making about these changes to antenatal care? Have you had any feedback from service users?
(6) Do you think any of these changes to remote antenatal care could have been improved?
(7) Did you create any local guidance? If so, please tell us about it.
(8) Have you noticed any impact from moving to remote antenatal care, and are these impacts being monitored?

(9) Do you have examples of what has worked well in remote antenatal care? What will you retain for future practice?
(10) What aspects of remote antenatal care don't work well or could be improved?
(11) How well do you feel that current remote antenatal care addresses the needs of pregnant women?
(12) Can you comment on how Black, Asian, or other Minority Ethnic groups and/or vulnerable women and families have been impacted?
(13) What are your thoughts about pregnant women doing some of their measurements at home as part of remote antenatal care?
(14) How well do you feel that current remote antenatal care addresses the needs of clinicians delivering care?
(15) What would you include in guidance for healthcare professionals for remote antenatal care?
(16) Is there anything else you wish to add about remote antenatal care that we have not discussed?

S2. Characteristics of the interview sample

	Service users N (%)		Health care professionals, other stakeholders N (%)		Managers and system-level stakeholders	
**Participants**	45		34		14	
**Gender**						
**Female**	45	(100)	31	(91)	11	(78)
**Male**	0	0	3	(9)	3	(22)
**Ethnicity**[1]						
**White**	24	(54)	N/A		N/A	
**Black**	9	(20)	N/A		N/A	
**Asian**	5	(11)	N/A		N/A	
**Mixed Ethnicity**	5	(11)	N/A		N/A	
**Other**	1	(2)	N/A		N/A	
**Did not say**	1	(2)	N/A		N/A	
**ONS Region**						
**Greater London**	22	(49)	5	(14.5)	7	(50)
**West Midlands**	3	(7)	5	(14.5)	1	(7)
**South East England**	1	(2)	5	(14.5)	1	(7)
**East Midlands**	4	(9)	1	(3)	0	(0)
**East of England**	3	(7)	5	(14.5)	1	(7)
**North East England**	0	(0)			0	(0)
**Scotland**	3	(7)			0	(0)
**South West England**	4	(9)	1	(3)	1	(7)
**Wales**	0	(0)	2	(6)	0	(0)
**North West England**	2	(4)	1	(3)	0	(0)
**Yorkshire and the Humber**	0	(0)	4	(12)	0	(0)
**Northern Ireland**	0	(0)	1	(3)	0	(0)
**Channel Islands**	0	(0)			0	(0)
**Did not say**	3	(7)	4	(12)	3	(21)
**Total number of interview participants**	93					

[1] As categorised by the ONS recommendations for country-specific ethnic group data collection https://www.ons.gov.uk/methodology/classificationsandstandards/measuringequality/ethnicgroupnationalidentityandreligion
